# Apoptotic and senolytic effects of hERG/Eag1 channel blockers in combination with temozolomide in human glioblastoma cells

**DOI:** 10.1007/s00210-025-03955-w

**Published:** 2025-03-24

**Authors:** Bodo Haas, Inken Roth, Luisa Säcker, Maria Wos-Maganga, Lea Beltzig, Bernd Kaina

**Affiliations:** 1 Federal Institute for Drugs and Medical Devices (BfArM), Kurt-Georg-Kiesinger-Allee 3, 53175 Bonn, Germany; 2 Faculty of Applied Natural Sciences, TH Köln - University of Applied Sciences, Campus Platz 1, 51379 Leverkusen, Germany; 3 Institute of Toxicology, University Medical Center, Obere Zahlbacher Strasse 67, 55131 Mainz, Germany

**Keywords:** Glioblastoma, Temozolomide, hERG channel, Eag1 channel, Chemosensitizer

## Abstract

Temozolomide (TMZ) concomitant with radiotherapy is the first-line treatment for glioblastoma. However, treatment resistance is frequently observed in patients. Cellular senescence (CSEN) induced by TMZ has been proposed to be one underlying mechanism resulting in resting cells, causing inflammation and possibly recurrences if senescent cells re-enter the cell cycle after treatment. Inhibition of the K^+^ channels human ether-à-go-go type 1 (Eag1) and human ether-à-go-go-related gene (hERG) has shown promising effects in several tumor types including glioblastoma through growth inhibition and induction of apoptosis. In the present study, we analyzed the impact of hERG/Eag1 inhibition on apoptosis and CSEN on its own and in combination with TMZ in a panel of human glioblastoma cell lines and primary glioblastoma cells. hERG/Eag1 protein expression was determined by Western blotting and immunocytochemistry. Cytotoxicity of astemizole and terfenadine alone or in combination with TMZ was assessed by MTT assays. Apoptotic yields were determined by Annexin V/propidium iodide staining, and CSEN was quantified by determining SA-β-galactosidase levels through flow cytometry. We observed a similar protein expression of hERG and Eag1 in all glioblastoma cell lines and primary glioblastoma cells. Astemizole and terfenadine were cytotoxic in glioblastoma cells at low micromolar concentrations (5–10 µM range) through induction of apoptosis. In combination with TMZ, both drugs synergistically sensitized glioblastoma cells to TMZ-induced apoptosis. Moreover, astemizole reduced significantly the TMZ-induced CSEN level, indicating its impact on CSEN induction. Here, we show for the first time that blocking hERG/Eag1 channels in glioblastoma cells can relief TMZ-induced CSEN and synergistically ameliorates cytotoxicity through the induction of apoptosis.

**Supplementary Information:**

The online version contains supplementary material available at 10.1007/s00210-025-03955-w.

## Introduction

Glioblastoma (WHO grade 4) is the most common malignant brain tumor in adulthood and often arises de novo without any clinical history (Komori 2022; Louis et al. 2021). The poor prognosis and the lack of effective therapies, including radiation and drug resistance, is devastating. Beside surgical resection and radiation, adjuvant and concomitant treatment with the alkylating drug temozolomide (TMZ) is the gold standard in glioblastoma therapy (Stupp et al. 2009; Stupp et al. 2005). However, TMZ prolongs the lifespan of patients by only 3 months; the 5-year survival rate is only 6%, and drug resistance is a remaining challenge (Price et al. 2024). All of this indicates that there is a high medical need for new treatment modalities.

We have previously reported that TMZ-resistance of glioblastoma cells might be a consequence of induction of a senescent cell population, which is characterized by resistance to TMZ-induced apoptosis, high level of residual DNA double-strand breaks (DSBs) and a sustained activation of the DNA damage response (DDR) (Aasland et al. 2019; Beltzig, et al. 2022b). Dormant senescent cells were shown to be able to re-enter the cell cycle after treatment cessation and are characterized by a proinflammatory phenotype, which further promotes tumor growth (Riviere-Cazaux et al. 2023; Zhang et al. 2023). Therefore, elimination of the senescent cell population has been suggested as a promising strategy to improve the outcome of glioblastoma therapy (Kaina 2023).

The two members of the KCNH family of voltage-gated potassium ion (K^+^) channels, human ether-à-go-go type 1 (Eag1 encoded by *KCNH1*) and human ether-à-go-go-related gene (hERG encoded by *KCNH2*), have been implicated in cancer development (Pardo and Stuhmer 2014; Wang et al. 2017) and hold promise as therapeutic targets (He et al. 2020; Wulff et al. 2009). While hERG is expressed in various tissues such as heart, brain, gastrointestinal tract, urinary bladder, and pancreas, Eag1 is mainly found in the brain (Martin et al. 2008; Vandenberg et al. 2012). However, both KCNH channels have also been shown to be overexpressed in several tumor types, including colon, breast, and cervical cancers, melanoma, neuroblastoma, and glioblastoma (Lastraioli et al. 2015; Meyer and Heinemann 1998; Patt et al. 2004). In patients, overexpression of hERG and Eag1 in glioblastoma results in poor prognosis and reduced survival (Martinez et al. 2015; Shugg et al. 2021; Zheng and Song 2023). KCNH expression is known to be linked to cell cycle phase and essential for cell cycle progression. Increased KCNH channel activity leads to increased proliferation (Pardo et al. 1999). Interestingly, activation of the hERG channel has been shown to activate a senescence program in breast cancer cells, highlighting its role also in senescence development (Lansu and Gentile 2013).

Despite this knowledge, the regulation of proliferation in tumor cells by K^+^ channels is not fully understood. It is known that overexpression of K^+^ channels in melanoma cells leads to hyperpolarization, which promotes the influx of calcium ions (Ca^2^^+^). Ca^2^^+^ is important for the transition from G1 to S phase in the cell cycle and, therefore, leads to increased proliferation (Nilius et al. 1993; Nilius and Wohlrab 1992). Another mechanism related to increased proliferation is the link between KCNH channels and cell volume. Cell shrinkage is accompanied by high expression of K^+^ channels, which deforms the cell and leads to changes in phosphatases and protein kinases, resulting in uncontrolled cell proliferation. Eag1 channel inhibitors keep the cell in G1/S phase and increase cell volume, both of which lead to a decreased proliferation rate (Wonderlin and Strobl 1996). Several studies indicate that inhibition of hERG and/or Eag1 channels with specific inhibitors in cultured glioblastoma cells reduces proliferation and induces apoptosis (Dong et al. 2023; Shugg et al. 2021; Staudacher et al. 2014). The hERG Inhibitor E-4031 was able to inhibit sphere formation in human glioblastoma xenografts in mice (Pointer et al. 2017). Along this line, suppression of Eag1 expression in glioblastoma cells resulted in growth inhibition and sensitization to drug treatment (Bai et al. 2013; Cunha et al. 2013; Sales et al. 2016). Consequently, inhibition of those channels has been proposed as promising treatment strategy for gliomas (Elias et al. 2023), which prompted us to further investigate hERG/Eag1 inhibition in glioblastoma cells in relation to apoptosis induction and impact on cellular senescence (CSEN).

Here, we aimed at disentangling the contribution of hERG/Eag1 inhibition to TMZ-induced cytotoxicity and CSEN, using established human glioblastoma cell lines and primary glioblastoma cells, by applying the potent inhibitors astemizole and terfenadine as model substances (Toplak et al. 2022). We measured concentration–response curves in 3-(4,5-dimethylthiazol-2-yl)−2,5-diphenyltetrazolium bromide (MTT) assays of astemizole and terfenadine either alone or in combination with TMZ, and in a panel of glioblastoma cells, quantified the effects and determined IC_50_ values. We show that astemizole and terfenadine are both cytotoxic to glioblastoma cells by induction of apoptosis. We also report that both drugs synergistically sensitize glioblastoma cells to TMZ, inducing a high yield of apoptosis and reducing the CSEN level.

## Materials and methods

### Compounds

Temozolomide, astemizole, and terfenadine were dissolved in dimethyl sulfoxide (DMSO). All compounds were purchased from Merck KGaA (Darmstadt, Germany).

### Cell culture

U87-MG, U251-MG, and U373-MG (Uppsala) human glioblastoma cell lines were obtained from Sigma-Aldrich (HPA Culture Collections, St. Louis, USA). The A172 and LN229 human glioblastoma cell line were obtained from American Type Culture Collection (ATCC, Manassas, USA). Cell lines were not used beyond passage 20. U251 Cells were cultivated in Roswell Park Memorial Institute (RPMI) 1640 medium containing 10% fetal calf serum (FCS; both from Biochrom, Berlin, Germany), 2 mM L-glutamine, 100 IU/ml penicillin, and 100 µg/ml streptomycin (all from Biowest, Nuaillé, France). Primary cells and all other cell lines were cultivated in Dulbecco’s Modified Eagle Medium (DMEM) containing 10% FCS, 2 mM L-glutamine, 100 IU/ml penicillin, and 100 µg/ml streptomycin (all from Biowest, Nuaillé, France). Glioblastoma cells and tissue specimens derived from a human glioblastoma tumor biopsy were obtained from the University Hospital Cologne and characterized as previously described (Haas et al. 2018).

### Western blot analysis

To prepare whole cell lysates, cells were washed with ice cold phosphate-buffered saline (PBS; Biowest, Nuaillé, France) and lysed with ice cold RIPA lysis buffer including 1 × HaltTM protease inhibitor cocktail (both from Thermo Fisher Scientific, Waltham, USA) incubated on ice for 30 min, and centrifuged for 20 min at 4 °C. The supernatant was used for protein content determination and subsequent immunoblotting. For immunoblotting, standard procedures using the following antibodies were used as previously described (Haas et al. 2009): anti-KCNH1 (APC-104; 1:250) and anti-KCNH2 (1:250; APC-109; both from Alomone Labs. Ltd., Jerusalem, Israel) combined with goat anti-rabbit Poly-HRP (1:10,000; Thermo Fisher Scientific, Waltham, USA) and β-Actin-HRP (C-4; Santa Cruz Biotechnology, Dallas, USA). Immunoblots were developed with ECL SuperSignal (Thermo Fisher Scientific, Waltham, USA) and densitometric analysis was performed using ImageJ software.

### MTT assay

For MTT assays, we followed a published protocol (Eckstein et al. 2009). Briefly, 5000 U251 cells, 7500 U87 and U373 cells, or 15,000 primary glioblastoma cells were plated on 96-wells and grown at 37 °C and 5% CO_2_ overnight. Cell survival after exposure to TMZ, astemizole or terfenadine as indicated was determined after 72 h. Final DMSO concentrations in media did not exceed 1%. Absorbance was measured with a Tecan Microplate Reader (Tecan, Hombrechtikon, Switzerland) at 590 nm.

### Fluorescence microscopy

150,000 cells were seeded in 2 mL culture medium in small glass bottom dishes (Willco Wells B.V., Amsterdam, Netherlands) and incubated at 37° C and 5% CO_2_ for 48 h. Thereafter, cells were washed twice with PBS and fixed with 4% paraformaldehyde/PBS for 10 min. Fixed cells were washed 3-times with PBS and blocked in 2% bovine serum albumin (BSA; Sigma-Aldrich, St. Louis, USA) including 0.2% Triton X-100 for 30 min. Thereafter cells were incubated with Anti-KCNH2-extracellular-FITC (APC-109-F; Alomone Labs Ltd., Jerusalem, Israel.) and Anti-KCNH1 (APC-104; Alomone Labs Ltd., Jerusalem, Israel) in 2% BSA (1:50) at 4° C overnight. The next day cells were washed and Anti-KCNH1 labelled probes were incubated with Anti-rabbit IgG (H + L) Alex Fluor 688 (Life Technologies) in 2% BSA (1:500) for 1 h. All probes were counterstained with NucRed Dead 647 ReadyProbes Reagent (Thermo Fisher Scientific, Waltham, USA) for 5 min. Finally, stained cells were washed for 3 times with PBS and covered with a coverslip by using fluorescent mounting medium (Merck KGaA, Darmstadt, Germany). Cells stained with secondary antibody only were used as negative control. Eag1/hERG expression was visualized by using a Zeiss LSM 780 microscope (Carl Zeiss AG, Oberkochen, Germany) and 40 × magnification.

### Annexin V apoptosis assay

For apoptosis measurements, the FITC Annexin V Apoptosis Detection Kit ll (BD Biosciences, Franklin Lakes, USA) was used according to the manufacturer´s protocol. Briefly, cells were seeded into 6-well plates and incubated at 37° C and 5% CO_2_ overnight. After compound treatment for 72 h or 120 h, cells were trypsinized and centrifuged for 4 min at 1500 × g. The supernatant was removed and cells were resuspended in 500 µL binding buffer. Five microliter propidium iodide (PI) and 5 µL Annexin V-FITC were mixed with 100 µL of cells in binding buffer. After 15 min of incubation on ice, samples were analyzed by flow cytometry using a FACSCalibur flow cytometer (Becton Dickinson GmbH, Heidelberg, Germany). The CellQuest pro software (Becton Dickinson GmbH, Heidelberg, Germany) was used for data analysis. Annexin V-/PI- cells were defined as living, annexin V + /PI- and annexin V + /PI + cells were defined as apoptotic cells, and annexin V-/PI + cells were defined as necrotic cells.

### Quantification of cellular senescence (CSEN)

The percentage of senescent cells within the population was determined by flow cytometry through the senescence-associated β-galactosidase, a marker for senescence, which was quantified by its reaction with the substrate C_12_FDG. To this end, cells were preincubated with 300 µM chloroquine (Merck KGaA, Darmstadt, Germany) for 30 min at 37 °C and 5% CO_2_ to inhibit endogenous β-galactosidase activity and kept in the dark from this point on. C_12_FDG (Abcam, Cambridge, UK) was added from a 20 mM stock solution to a final concentration of 33 µM to each sample, and cells were incubated for an additional 90 min at 37° C and 5% CO_2_. Cells were then collected, washed, resuspended in an adjusted amount of PBS, and stored on ice until measurement. The FACS Canto II flow cytometer (Becton Dickinson GmbH, Heidelberg, Germany) was used for data acquisition using Flowing Software 2.5.1 program (Perttu Terho, Turku Center for Biotechnology, Turku, Finland) for data analysis. Proliferating cells served as control in each experiment as described (Beltzig et al. 2022a).

### Data analysis and statistical methods

Concentration effect curves of MTT experiments were fitted to data points by nonlinear regression analysis using the four-parameter logistic equation. Top was set to 100% and bottom to 0% viability (GraphPad Prism, La Jolla, USA). Statistical differences between several groups were determined by ANOVA followed by Tukey’s post-hoc test. Data are presented as mean ± SEM. To analyze drug combination effects, the Coefficient of Drug Interaction (CDI) was calculated according to the following equation: $CDI= \frac{A+B-A\times B}{AB}$

When the CDI is under, above or equal to 1, this is indicative of synergy, antagonism or additivity, respectively (Foucquier and Guedj 2015).

## Results

### Expression analysis of KCNH channels in glioblastoma cells and tissue

**Fig. 1:**
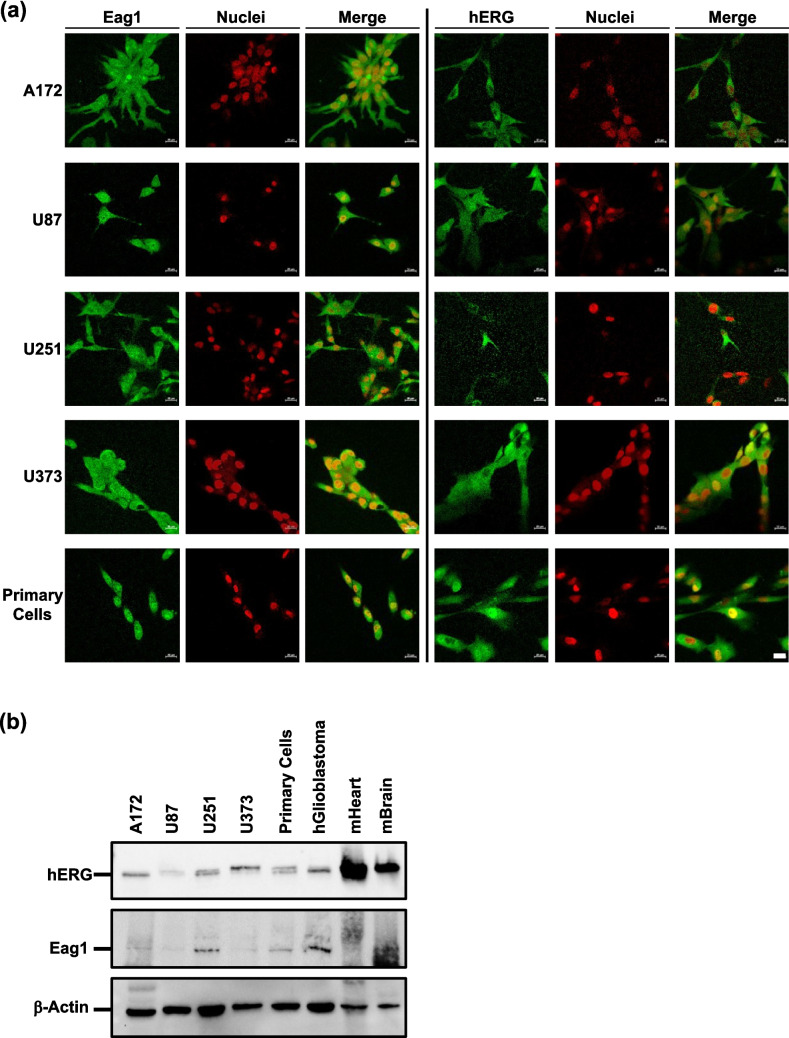
KCNH protein expression analysis in human glioblastoma cells and tissue. (**a**) Representative immune fluorescence images showing expression of hERG and Eag1 (green fluorescence) in human A172, U87, U251, U373 glioblastoma cell lines and primary human glioblastoma cells. Nuclei are stained in red; scale bar, 20 µm. (**b**) Representative Western blot showing protein expression of hERG and Eag1 in whole cell lysates of human A172, U87, U251, U373 glioblastoma cell lines, primary human glioblastoma cells and human glioblastoma tissue (hGlioblastoma). Murine heart (mHeart) and brain (mBrain) tissues were used as positive controls. β-actin Western blots were performed to control for loading

Both expression of O^6^-methylguanine-DNA methyltransferase (MGMT) and p53 mutations have been linked to TMZ sensitivity of glioblastoma cell lines (Hermisson et al. 2006; Lee 2016). In our study we used cell lines with undetectable MGMT expression. U87, LN229 and A172 are functional p53 wildtype while p53 is mutated in U373 and U251 cells. The mutation status of all used cells can be found in Suppl. Table 1. The expression of hERG and Eag1 in glioblastoma cells and tissues has previously been reported (Masi et al. 2005; Patt et al. 2004). To prove if both channels are actually expressed under the experimental conditions, we determined the protein expression in our panel of glioblastoma cell lines and primary glioblastoma cells. As shown in Fig. 1, hERG and Eag1 channels are expressed to a similar extent in four glioblastoma cell lines (A172, U87, U251, U373) and primary glioblastoma cells, determined by both immunocytochemistry (Fig. 1a) and Western blotting (Fig. 1b and Suppl. Figure 1 a and b). In addition, we confirmed expression of hERG and Eag1 in glioblastoma tissue isolated from a patient biopsy (Fig. 1b).

### Blocking hERG/Eag1 induces cytotoxicity in human glioblastoma cells

**Fig. 2:**
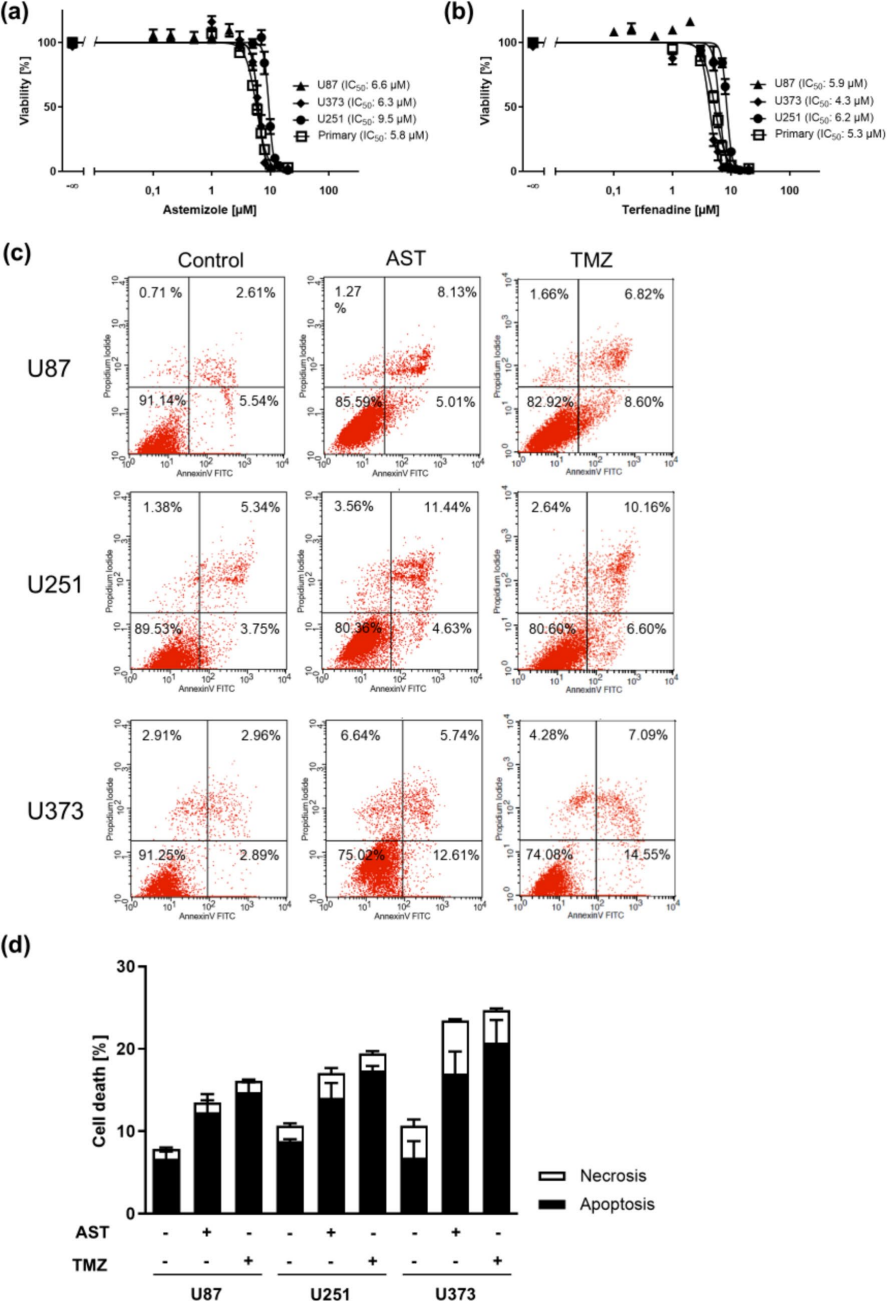
Cytotoxic effects of astemizole and terfenadine in glioblastoma cells. 3-(4,5-dimethylthiazol-2-yl)−2,5-diphenyltetrazolium bromide (MTT) assays of three human glioblastoma cell lines and primary glioblastoma cells treated with increasing concentrations of astemizole (**a**) and terfenadine (**b**) for 72 h, as indicated. (**c**) Representative AnnexinV/propidium iodide (PI) FACS analysis of glioblastoma cell lines U87, U251, and U373 treated with the respective astemizole (AST) IC_50_ or 1 mM temozolomide (TMZ) for 72 h. (**d**) Percent cell death induced via apoptosis or necrosis after AST and TMZ treatment. Annexin V positive cells were considered apoptotic and PI positive/Annexin V negative cells necrotic. Values are displayed as mean ± SEM (*n* = 3–4)

We next investigated whether inhibition of hERG/Eag1 channels with the well-established and highly potent inhibitors astemizole and terfenadine (Toplak et al. 2022) induces cytotoxicity in selected glioblastoma cells in MTT experiments. We recorded very similar IC_50_ values in all cell lines in the range from 4 to 9 µM after 72 h treatment (Fig. 2a and b). Both drugs displayed a very steep concentration response in all cell lines (Hill slopes ranging from −5.02 to −5.37, Suppl. Table 2). The lowest IC_50_ of 5.8 µM for astemizole was obtained for primary glioblastoma cells (Fig. 2a) and the lowest IC_50_ of 4.8 µM for terfenadine in U373 cells (Fig. 2b). U251 appeared to be the most resistant cell line to both drugs (IC_50s_ of 9.5 µM and 6.2 µM for astemizole and terfenadine, respectively, Fig. 2a and b). In order to test if cell death is driven by apoptosis or necrosis, we performed Annexin V/PI staining followed by flow cytometry of selected cell lines treated with astemizole concentrations close to the previously determined IC_50_ values in comparison to TMZ. Representative plots are shown in Fig. 2c. The experiments revealed that > 70% of cell deaths accounted for apoptosis (Fig. 2d).

### Astemizole and terfenadine sensitize human glioblastoma cells to TMZ treatment

**Fig. 3:**
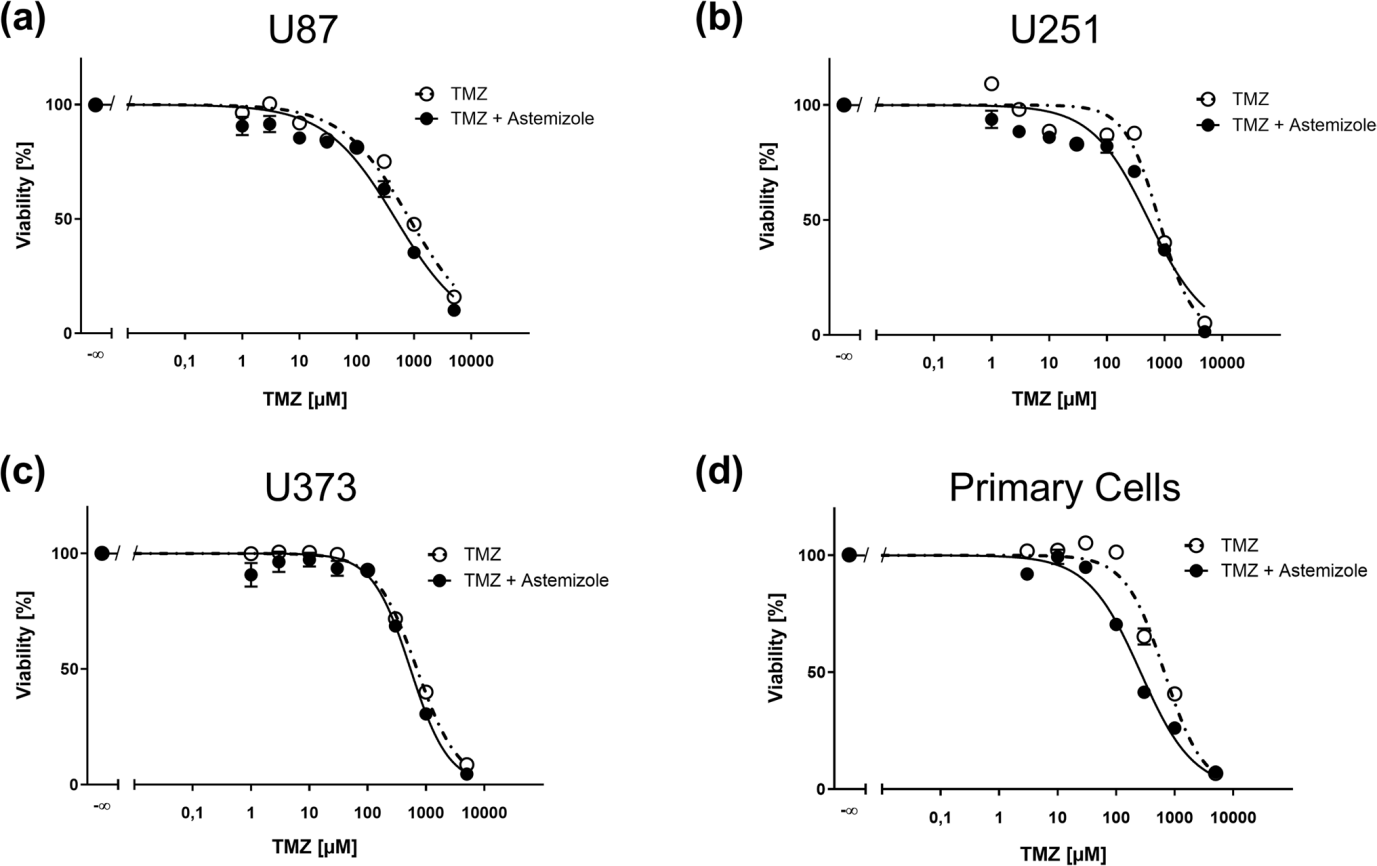
Astemizole sensitizes glioblastoma cells to temozolomide (TMZ) treatment. MTT assays of U87 (**a**), U251 (**b**), U373 (**c**), and primary glioblastoma cells (**d**) treated with increasing concentrations of TMZ for 72 h alone or in combination with astemizole (5–7 µM). Values are displayed as mean ± SEM (*n* = 3)

It has been reported that astemizole was capable of sensitizing U87 cells to TMZ when given at fixed concentrations at a 72 h treatment schedule (Sales et al. 2016). To determine a concentration relationship in a panel of cell lines, we treated three different glioblastoma cell lines and primary glioblastoma cells with ascending TMZ concentrations combined with respective astemizole concentrations close to the IC_50_ values obtained in previous experiments and performed MTT assays. In addition, we also treated primary cells with TMZ and terfenadine using the same experimental set up. We observed left-shifts of the MTT concentration–response curves after astemizole/TMZ co-treatment and a reduction in TMZ IC_50_ values in all cell lines and primary glioblastoma cells (Fig. 3a–d), indicative of a sensitization. The respective IC_50_ values are displayed in Table 1.

**Table 1:** Temozolomide (TMZ) IC_50_ values derived from MTT assays of TMZ treated (Ctr.) glioblastoma cells with or without astemizole (AST) or terfenadine (TERF). Values are displayed as mean (*n* = 3). The 95% confidence interval (CI) is shown in brackets. ND, not determined

	TMZ IC_50_ (95% CI), [µM]			
Cells	U87	U251	U373	Primary
Ctr	836 (713 – 980)	805 (694 – 935)	700 (637 – 769)	584 (525 – 661)
AST	476 (384 – 590)	533 (434 – 656)	542 (467 – 628)	261 (231 – 294)
TERF	ND	ND	ND	188 (144 – 244)

While the observed reduction in TMZ IC_50_ values after co-treatment was low in U251 and U373 cells, the TMZ IC_50_ was approximately reduced by twofold in U87 and primary glioblastoma cells. A similar strong sensitization was observed with terfenadine in primary glioblastoma cells (Suppl. Figure 2a and Table 1).

### hERG/Eag1 inhibition synergistically increases TMZ-induced apoptosis

**Fig. 4:**
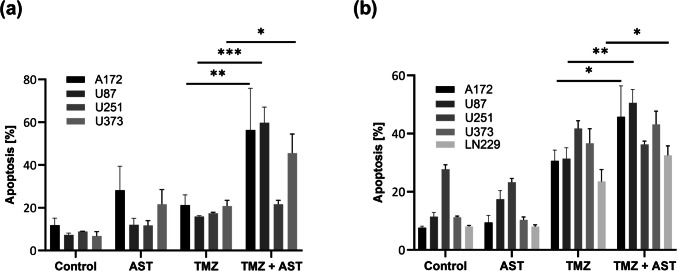
Sensitization of glioblastoma cells to temozolomide (TMZ)-induced apoptosis by astemizole (AST) at different treatment schedules. AnnexinV/propidium iodide FACS analysis of glioblastoma cell lines treated with a high TMZ concentration (1 mM) for 72 h alone or in combination with astemizole (5–7 µM) (**a**), or a low TMZ concentration (50 µM) for 120 h alone or in combination with 2 µM astemizole (**b**). Values are displayed as mean ± SEM (*n* = 3); *, *p* < 0.05, ** *p* < 0.01, *** *p* < 0.001. One-way Anova with Tukey’s post-hoc test

We extended the experiment of TMZ and subsequent astemizole treatment by determining the apoptotic rates in several glioblastoma cell lines. Treatment with an astemizole IC_50_ and a fixed TMZ concentration (1 mM) significantly enhanced TMZ-induced apoptosis induction by approximately two- to threefold, with the exception of U251 cells, where no effect of astemizole was detected (Fig. 4a). Importantly, the coefficient of drug interaction (CDI) was < 1 for the A172, U87 and U373 cell lines, which indicates a synergistic effect (Table 2, column Apoptosis, TMZ high, 72 h).

**Table 2:** Coefficient of drug interaction (CDI) for apoptosis and cellular senescence (CSEN) of temozolomide (TMZ) and astemizole co-treatment at different time points and TMZ concentrations in various glioblastoma cell lines. ND, not determined

Coefficient of drug interaction (CDI)			
	Apoptosis		CSEN
***Cells***	***TMZ high, 72 h***	***TMZ low, 120 h***	***TMZ low,*** ***120 h***
A172	0.86	0.81	1.89
LN229	ND	0.93	2.18
U87	0.86	0.46	ND
U251	1.25	1.52	ND
U373	0.83	0.91	ND

Similar synergistic effects were observed in U87 cells treated with terfenadine and TMZ (CDI < 0.60; Suppl. Figure 2b). While astemizole and terfenadine induce apoptosis early (i.e., 2–3 days) after treatment, TMZ needs a much longer time for lesion processing and cell death pathway induction (5 to 8 days in glioblastoma cells, depending on the respective cell cycle progression) (He and Kaina 2019). Consequently, much lower and physiologically more relevant TMZ concentrations can be applied to effectively kill glioblastoma cells. Therefore, we repeated the experiment with an extended panel of glioblastoma cell lines and treated for 120 h with 50 µM TMZ. This occurred without and with a low astemizole concentration (2 µM) that was not yet cytotoxic when applied alone. In A172, U87 and LN229, but not U251 and U373, we observed a statistically significant and synergistic (CDIs < 1) effect of astemizole on TMZ-induced apoptosis levels (Fig. 4b and Table 2, column Apoptosis, TMZ low, 120 h).

### Astemizole reduces TMZ-induced CSEN

**Fig. 5:**
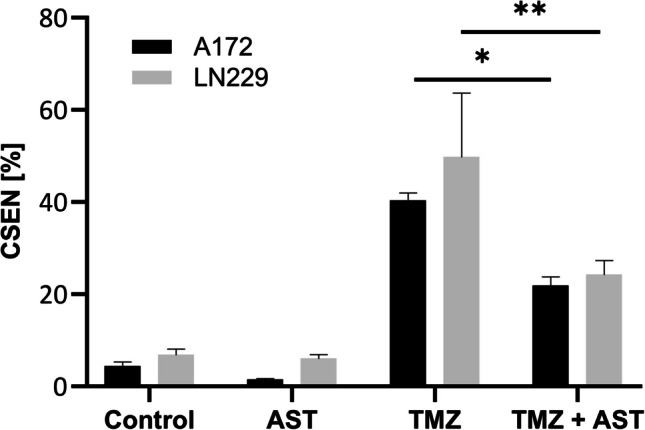
Effect of astemizole (AST) on temozolomide (TMZ)-induced cellular senescence (CSEN) in two glioblastoma cell lines. C_12_FDG analysis of LN229 and A172 cells treated with 20 µM TMZ for 120 h alone or in combination with 2 µM astemizole. Values are displayed as mean ± SEM of at least three independent experiments; *, *p* < 0.05, ** *p* < 0.01. One-way Anova with Tukey’s post-hoc test

Previously, we have shown that TMZ induces CSEN in functionally p53 wildtype LN229 and A172 glioblastoma cells, with a yield of up to 80% over time (Beltzig et al. 2022b). As senescent cells exhibit a proinflammatory phenotype, which is discussed to promote tumor growth (Aasland et al. 2019), strategies are desired to prevent or reduce the level of CSEN induction. To address if Eag1/hERG inhibition impacts CSEN, we determined the senescence level of A172 and LN229 cells following treatment with TMZ alone or in combination with astemizole for 120 h. As previously reported (Aasland et al. 2019; Beltzig et al. 2022b), TMZ significantly induced senescence in both cell lines, while astemizole (2 µM) was ineffective. Interestingly, when TMZ and astemizole were given concomitantly, the senescent cell population was significantly reduced by approximately 50% in both cell lines (Fig. 5 and Suppl. Figure 3). This was reflected by an CDI > 1 indicative of an antagonistic effect of astemizole on TMZ-induced CSEN (Table 2, column CSEN, TMZ low, 120 h).

## Discussion

A hallmark of glioblastoma is resistance to radio- and chemotherapy resulting in dismal prognosis. This is also reflected by the fact that about 70% of glioblastoma patients do not show a benefit following TMZ, which stresses the need to further explore mechanisms to enhance the cytotoxic activity of the drug (Chamberlain 2010). In this context it is of interest that the KCNH channels, hERG and Eag1, have been reported to impact glioblastoma proliferation, apoptosis and progression (Elias et al. 2023; He et al. 2020). Moreover, overexpression of both channels in glioma tissues was shown to be correlated with reduced survival and poor prognosis (Martinez et al. 2015; Shugg et al. 2021; Zheng and Song 2023). While several studies revealed that blocking hERG or Eag1 with inhibitors or a genetic knock-down induces cell cycle arrest and apoptosis in glioblastoma cells (Elias et al. 2023), only one study has proposed a sensitizing potential of Eag1 blockade to TMZ treatment in U87 cells (Sales et al. 2016). This prompted us to quantitatively investigate the chemosensitizing effects of hERG/Eag1 inhibition in a panel of human glioblastoma cell lines and patient-derived primary glioblastoma cells. We found that both channels were similarly expressed in all the cells rendering them suitable for further investigations.

Initially, we tested the two hERG/Eag1 blocker astemizole and terfenadine alone and found that both are cytotoxic in all glioblastoma cells to a similar extent (IC_50_ values for both, astemizole and terfenadine, were in the range of 5–8 µM after 72 h-treatment). Cytotoxicity was related to the induction of apoptosis (> 70%), which appeared to be the main cause of cell death. This is in line with what was reported after hERG/Eag1 inhibition in other tumor cell lines (Jehle et al. 2011). As both compounds already induced cytotoxicity after 72 h, we initially used this time to investigate a sensitizing potential on TMZ treatment in concentration–response experiments. Astemizole and terfenadine induced a systematic left shift of the concentration–response curves and reduced the TMZ IC_50_ by around twofold in U87 and primary human glioblastoma cells indicative of a strong sensitizing effect across various TMZ concentrations. In U251 and U373 cells, the concentration–response curves largely overlapped and reductions in TMZ IC_50_ values after co-treatment were smaller. In terms of apoptosis induction synergistic effects of astemizole on TMZ treatment at different experimental conditions (e.g., high concentrations for 72 h vs. low concentrations for 120 h) were observed in most cell lines. Again, the U251 cell appeared to be non-responsive towards astemizole treatment in combination with TMZ (CDI > 1). The reasons for these differences remain speculative as all cell lines express hERG and Eag1 channels. But differences might be related to the mutation patterns of the cells. While all used cell lines are IDH wild-type and the MGMT promoter is methylated (Haas et al. 2018; Perazzoli et al. 2015), they differ in the p53 mutation status. U87, A172 and LN229 cells are functionally p53 wildtype and displayed a good response towards combination treatment. In contrast, U251 and U373 cells are p53-mutated (Beltzig et al. 2022b; Hermisson et al. 2006; Lee et al. 2020) and had a lower response rate. This suggests that the p53 status plays a role in the sensitivity of glioblastoma cells towards hERG/Eag1 inhibition, which needs however further and more detailed mechanistic studies. 

Recent studies revealed that glioblastoma cells respond to TMZ treatment with the induction of senescence, a phenomenon observed in many cancers upon treatment and described as therapy-induced senescence (TIS). TMZ-induced senescent cells go into a mitotic arrest and display an altered transcriptional, metabolic and secretory phenotype (Aasland et al. 2019; Beltzig et al. 2022b; Tchkonia et al. 2013). Moreover, it was proposed that CSEN might be a driver of tumor recurrence as senescent cells could re-enter the cell cycle after termination of treatment (Milanovic et al. 2018; Ouchi et al. 2016). Furthermore, senescent cells are characterized by a proinflammatory phenotype, which favors tumor growth (Aasland et al. 2019). Novel approaches aim at targeting CSEN by senolytic agents, and for glioblastoma some compounds have been identified so far (Riviere-Cazaux et al. 2023). An alternative strategy rests on inhibition of CSEN induction. TMZ is highly effective in inducing CSEN. Thus, the level of CSEN is raising 3 days after treatment and 8 days later about 80% of the population is in the senescent state (Beltzig et al. 2022b). In this context it is also interesting to note that hERG activation has been implicated in the induction of CSEN in breast cancer cells (Lansu and Gentile 2013). Here, we show for the first time that inhibition of hERG/Eag1 by concomitant treatment of glioblastoma cells with TMZ and astemizole significantly reduced TMZ-induced CSEN (by about 50%). This was accompanied by an increase in the apoptotic cell death level, which indicates that hERG/Eag1 inhibition has the potential to inhibit the CSEN pathway and pushing cells into the death pathway. We did not mechanistically follow up on our findings and clearly further research is needed to investigate the underlying mechanisms. However, our results are encouraging and might pave the way for further experimentation. Importantly, studies have shown that astemizole has good brain permeability (Di et al. 2003; Rojo et al. 2010) and, therefore, is proposed as treatment modality in Alzheimer’s and Parkinson’s disease (Rojo et al. 2010; Sun et al. 2016). One shortcoming of many hERG blocking agents like astemizole and terfenadine is their proarrhythmic and torsadogenic potential by the inhibition of hERG channels in cardiomyocytes (Zunkler 2006). But there are several other commercial drugs available (e.g., amitriptyline, haloperidol or fluoxetine) with good hERG/Eag1 blocking activity, but rather moderate proarrhythmic effects in patients. Most importantly, glioblastoma patients with a high hERG expression level who took those drugs in conjunction with TMZ/radiation had a better survival rate (Pointer et al. 2017).

Overall, we conclude that blocking hERG/Eag1 channels in glioblastoma is a promising strategy to overcome TMZ-induced senescence and drug resistance. Further in vitro and in vivo studies are required to define the mode of action and proof the efficacy in animal models before clinical use can be envisioned.

## Supplementary Information

Below is the link to the electronic supplementary material.

ESM 1 (PNG 167 KB)

High Resolution Image (TIF 1.51 MB)

ESM 2 (PNG 314 KB)

High Resolution Image (TIF 1.76 MB)

ESM 3 (PNG 1.24 MB)

High Resolution Image (TIF 6.34 MB)

Supplementary file4 (PDF 102 KB)

Supplementary file5 (PDF 79 KB)

## Data Availability

All source data for this work (or generated in this study) are available upon reasonable request.

## Declarations

### Disclaimer

The opinions mentioned throughout the following article are personal views of the authors and do not reflect an official position of the Federal Institute for Drugs and Medical Devices (BfArM).
