# Supplementary figures and images for: Apoptotic and senolytic effects of hERG/Eag1 channel blockers in combination with temozolomide in human glioblastoma cells
Source: Naunyn Schmiedebergs Arch Pharmacol. 2025 Mar 24;398(9):12267–78. doi: 10.1007/s00210-025-03955-w (PMC12449327; doi:10.1007/s00210-025-03955-w)

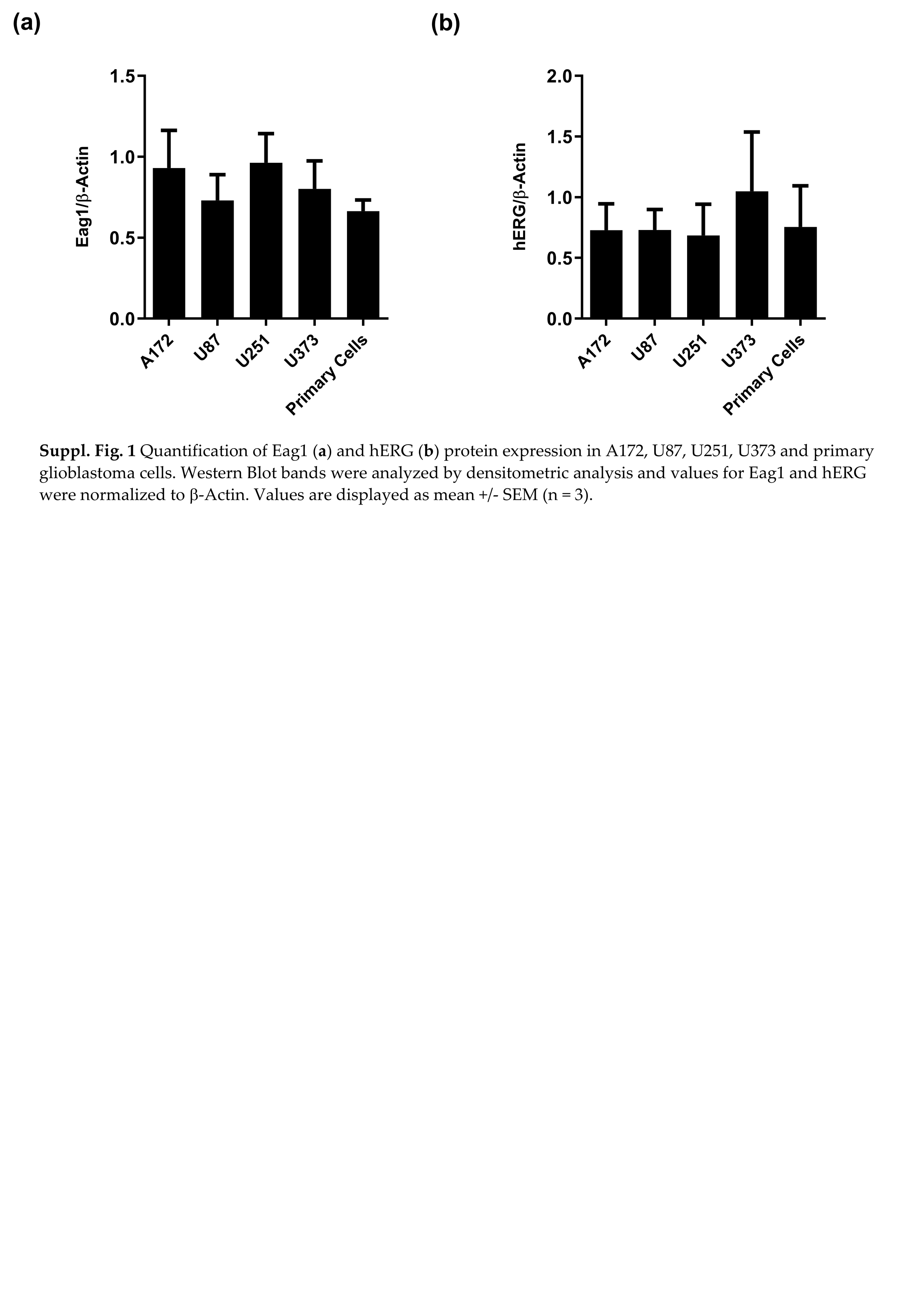

Supplement: Supplementary file 1 — (PNG 167 KB) [file 210_2025_3955_Fig6_ESM.png]

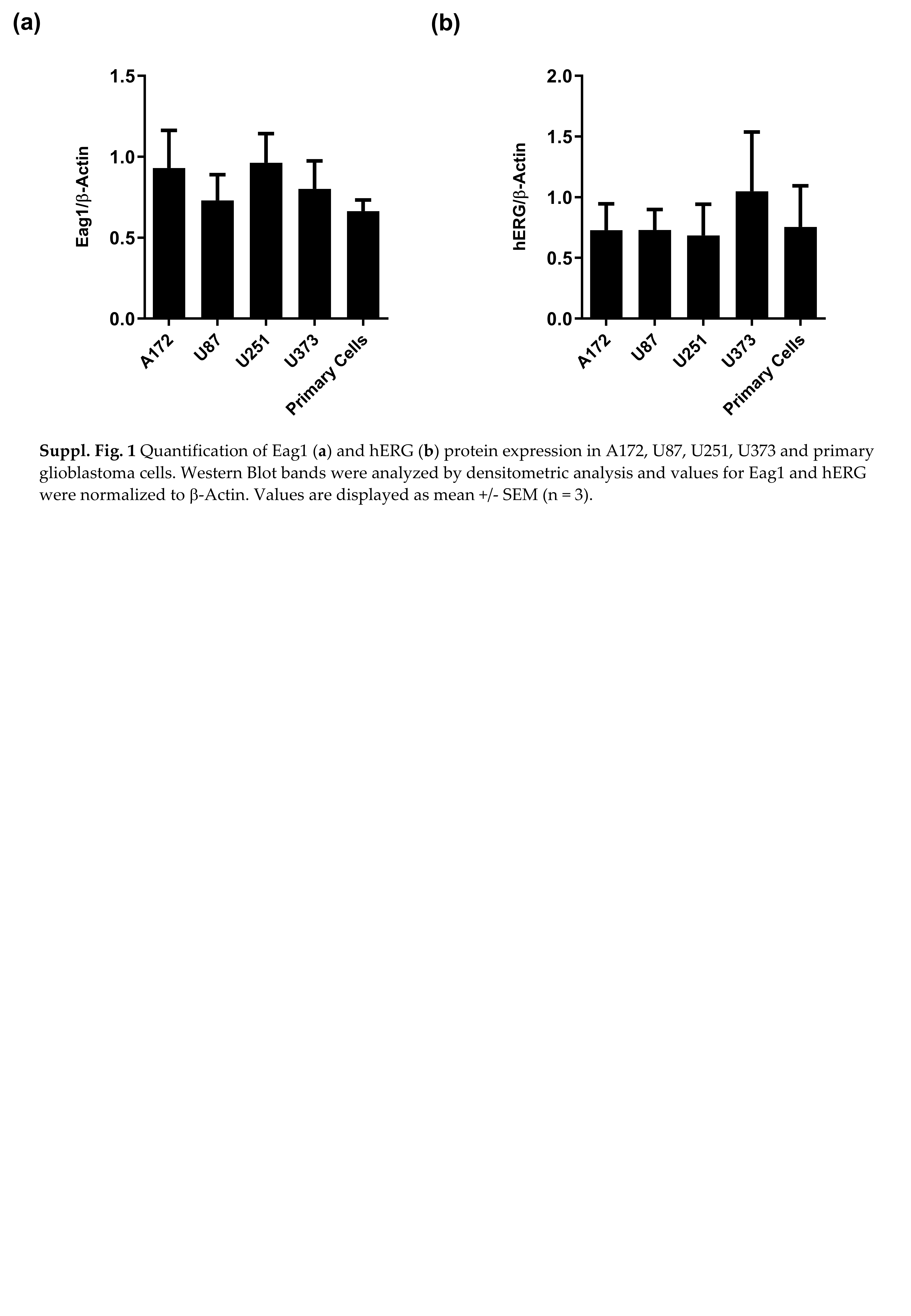

Supplement: Supplementary file 2 — High Resolution Image (TIF 1.51 MB) [file 210_2025_3955_MOESM1_ESM.tif]

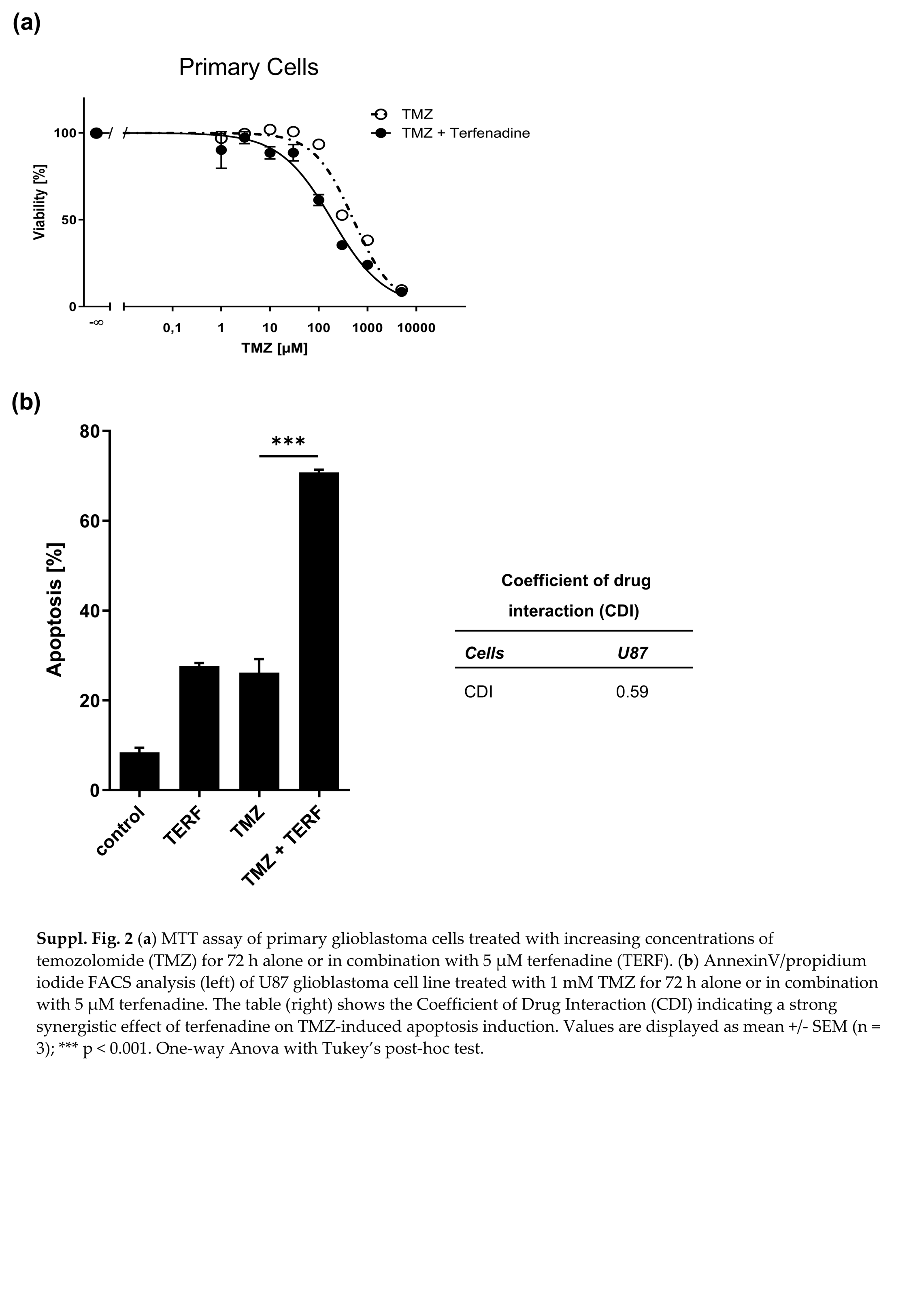

Supplement: Supplementary file 3 — (PNG 314 KB) [file 210_2025_3955_Fig7_ESM.png]

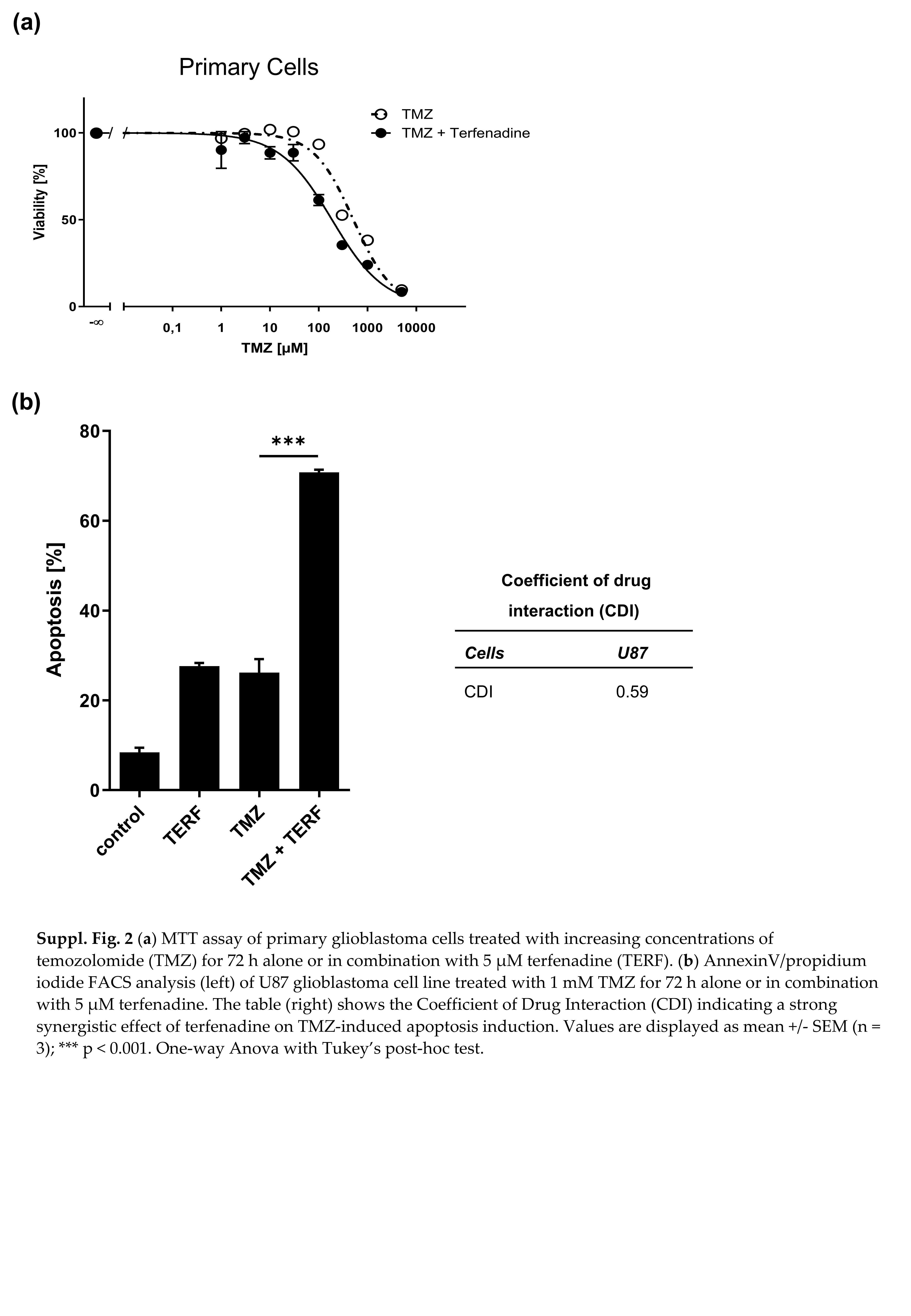

Supplement: Supplementary file 4 — High Resolution Image (TIF 1.76 MB) [file 210_2025_3955_MOESM2_ESM.tif]

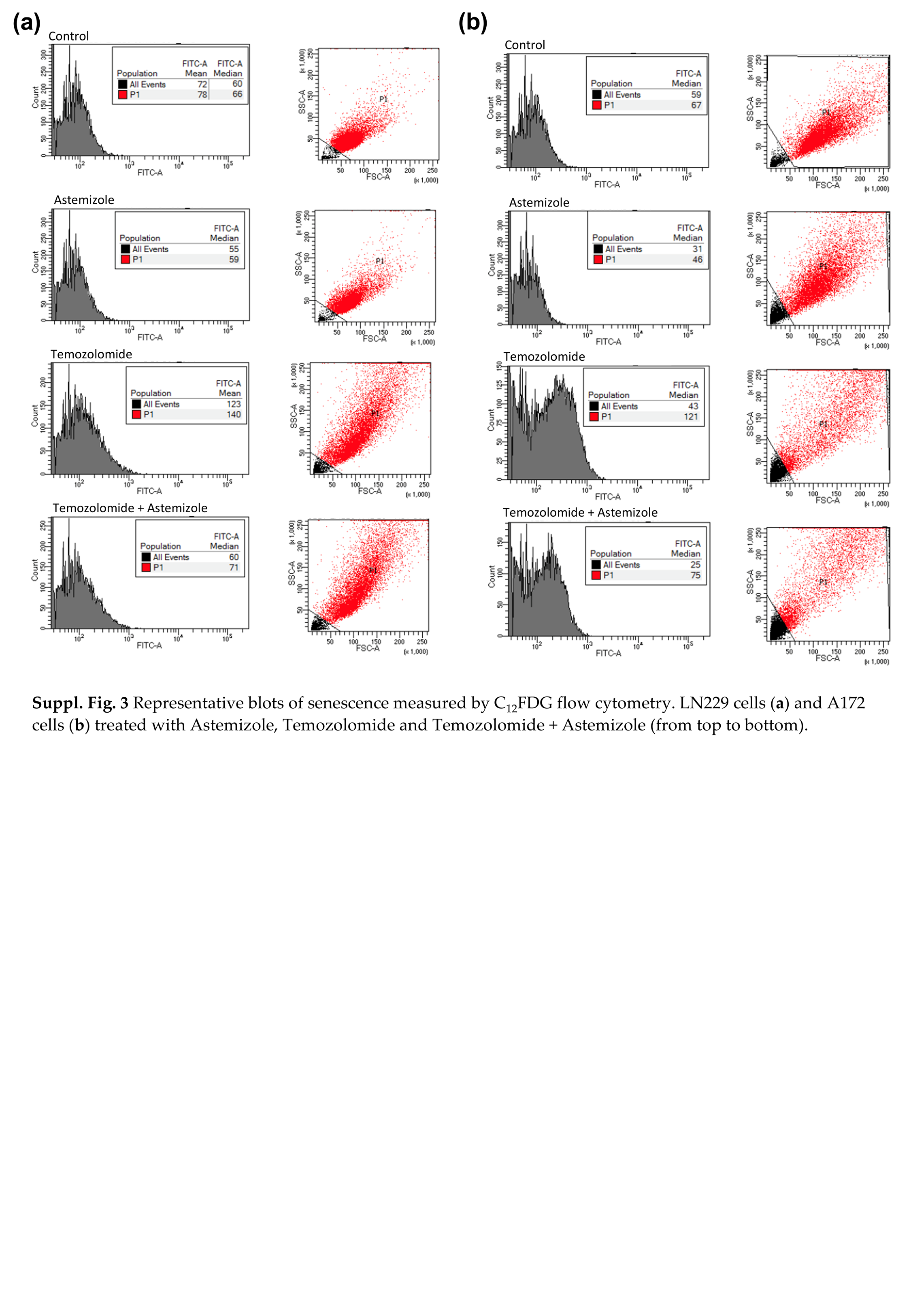

Supplement: Supplementary file 5 — (PNG 1.24 MB) [file 210_2025_3955_Fig8_ESM.png]

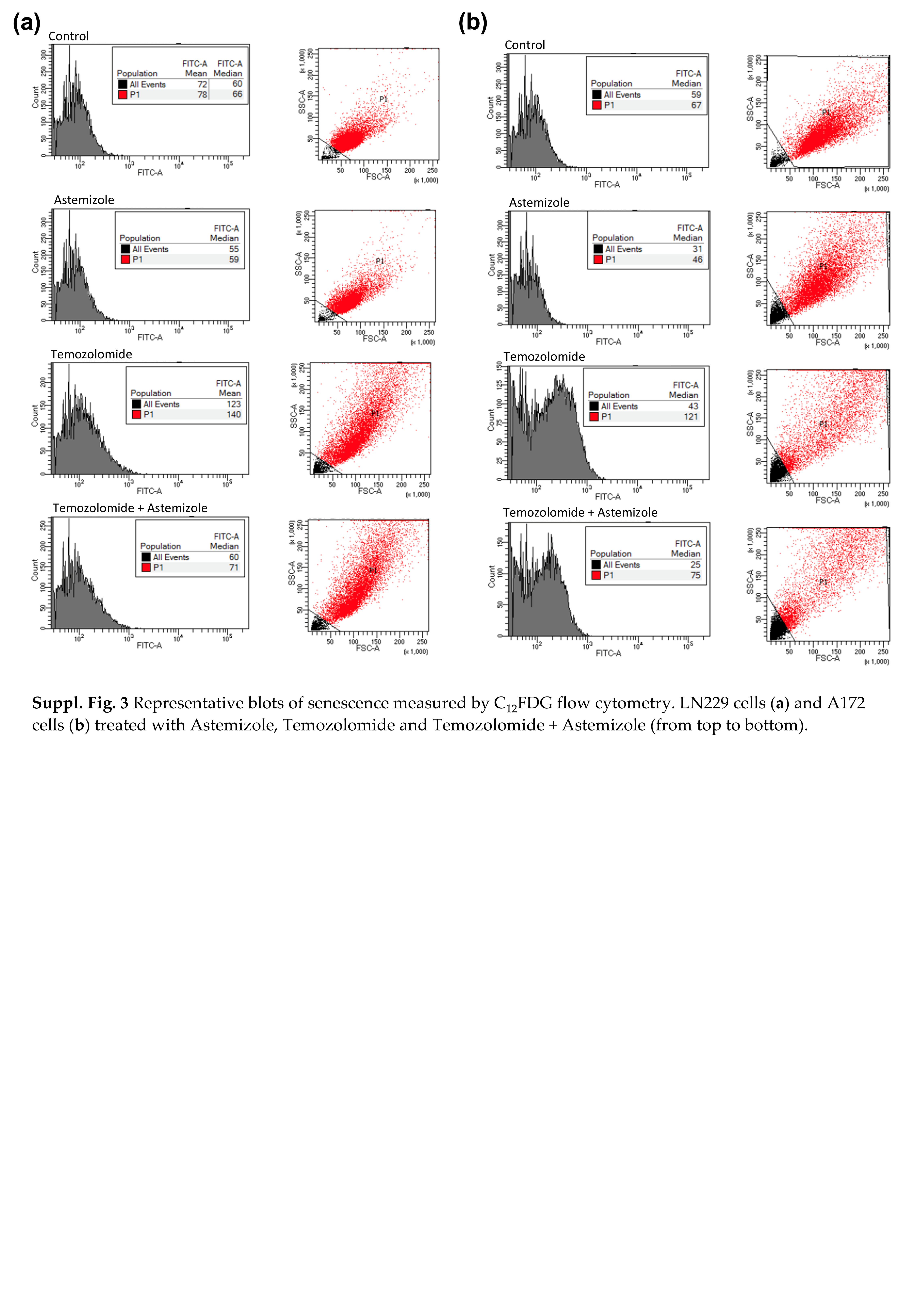

Supplement: Supplementary file 6 — High Resolution Image (TIF 6.34 MB) [file 210_2025_3955_MOESM3_ESM.tif]
