# Supplementary material for: Apoptotic and senolytic effects of hERG/Eag1 channel blockers in combination with temozolomide in human glioblastoma cells
Source: Naunyn Schmiedebergs Arch Pharmacol. 2025 Mar 24;398(9):12267–78. doi: 10.1007/s00210-025-03955-w (PMC12449327; doi:10.1007/s00210-025-03955-w)
Supplement: Supplementary file 7 — Supplementary file4 (PDF 102 KB) [file 210_2025_3955_MOESM4_ESM.pdf]

Supplementary Table 1: Genetic status of investigated cells.

Cells	p53	IDH	MGMT promoter status	PTEN
LN229	wt	wt	methylated	wt
A172	wt	wt	methylated	mut
U251	mut	wt	methylated	mut
U373	mut	wt	methylated	mut
U87	wt	wt	methylated	mut
Primary cells	unknown	wt	methylated	unknown

Abbreviations: IDH, isocitrate dehydrogenase; MGMT, O^6-methylguanine-DNA methyltransferase, PTEN, phosphatase and tensin homolog; Sources: Beltzig et al. 2022; Furnari et al. 1997; Haas et al. 2018; Hermisson et al. 2006; Lee et al. 2020; Perazzoli et al. 2015; American Type Culture Collection (https://www.atcc.org/)

References

Beltzig L et al. (2022) Senescence Is the Main Trait Induced by Temozolomide in Glioblastoma Cells Cancers (Basel) 14 doi:10.3390/cancers14092233

Furnari FB, Lin H, Huang HS, Cavenee WK (1997) Growth suppression of glioma cells by PTEN requires a functional phosphatase catalytic domain Proc Natl Acad Sci U S A 94:12479-12484 doi:10.1073/pnas.94.23.12479

Haas B et al. (2018) Inhibition of the PI3K but not the MEK/ERK pathway sensitizes human glioma cells to alkylating drugs Cancer Cell Int 18:69 doi:10.1186/s12935-018-0565-4

Hermisson M et al. (2006) O6-methylguanine DNA methyltransferase and p53 status predict temozolomide sensitivity in human malignant glioma cells J Neurochem 96:766-776 doi:10.1111/j.1471-4159.2005.03583.x

Lee YJ, Seo HW, Baek JH, Lim SH, Hwang SG, Kim EH (2020) Gene expression profiling of glioblastoma cell lines depending on TP53 status after tumor-treating fields (TTFields) treatment Sci Rep 10:12272 doi:10.1038/s41598-020-68473-6

Perazzoli G et al. (2015) Temozolomide Resistance in Glioblastoma Cell Lines: Implication of MGMT, MMR, P-Glycoprotein and CD133 Expression PLoS One 10:e0140131 doi:10.1371/journal.pone.0140131
