# Supplementary material for: Apoptotic and senolytic effects of hERG/Eag1 channel blockers in combination with temozolomide in human glioblastoma cells
Source: Naunyn Schmiedebergs Arch Pharmacol. 2025 Mar 24;398(9):12267–78. doi: 10.1007/s00210-025-03955-w (PMC12449327; doi:10.1007/s00210-025-03955-w)
Supplement: Supplementary file 8 — Supplementary file5 (PDF 79 KB) [file 210_2025_3955_MOESM5_ESM.pdf]

Supplementary Table 2: Hill slopes and IC_{50} values from MTT assays of glioblastoma cells treated with astemizole or terfenadine for 72 h.

	Astemizole			Terfenadine		
	**Hill slope**	**Log IC_{50}**	**IC_{50} [µM]**	**Hill slope**	**Log IC_{50}**	**IC_{50} [µM]**
**U87**	- 12.79 +/- 1.622	- 5.182 +/- 0.00503	6.6	- 22.54 +/- 6.856	- 5.23 +/- 0.00442	5.9
**U251**	- 10.61 +/- 1.395	- 5.02 +/- 0.00672	9.5	- 12.71 +/- 1.471	- 5.21 +/- 0.00428	6.2
**U373**	- 7.94 +/- 1.176	- 5.20 +/- 0.00852	6.3	- 6.156 +/- 0.744	- 5.37 +/- 0.0132	4.3
**Primary Cells**	- 4.689 +/- 0.3013	- 5.236 +/- 0.00548	5.8	- 4.434 +/- 0.4086	- 5.279 +/- 0.00912	5.3
